# Next-generation sequencing of small RNAs from inner ear sensory epithelium identifies microRNAs and defines regulatory pathways

**DOI:** 10.1186/1471-2164-15-484

**Published:** 2014-06-18

**Authors:** Anya Rudnicki, Ofer Isakov, Kathy Ushakov, Shaked Shivatzki, Inbal Weiss, Lilach M Friedman, Noam Shomron, Karen B Avraham

**Affiliations:** Department of Human Molecular Genetics and Biochemistry, Sackler Faculty of Medicine and Sagol School of Neuroscience, Tel Aviv University, Tel Aviv, 69978 Israel; Department of Cell and Developmental Biology, Sackler Faculty of Medicine and Sagol School of Neuroscience, Tel Aviv University, Tel Aviv, 69978 Israel; Clalit Health Services, Migdal HaEmek Clinic, Tel Aviv, Israel

**Keywords:** Deafness, Inner ear, Sensory epithelia, RNA-seq, MicroRNAs

## Abstract

**Background:**

The mammalian inner ear contains sensory organs, the organ of Corti in the cochlea and cristae and maculae in the vestibule, with each comprised of patterned sensory epithelia that are responsible for hearing and balance. The development, cell fate, patterning, and innervation of both the sensory and nonsensory regions of the inner ear are governed by tight regulation involving, among others, transcription factors and microRNAs (miRNAs). In humans, mutations in specific miRNA genes are associated with hearing loss. In mice, experimental reduction or mutations of miRNAs in the inner ear leads to severe developmental and structural abnormalities. A comprehensive identification of miRNAs in the sensory epithelia and their gene targets will enable pathways of auditory and vestibular function to be defined.

**Results:**

In this study, we used Next-Generation Sequencing (NGS) to identify the most prominent miRNAs in the inner ear and to define miRNA-target pairs that form pathways crucial for the function of the sensory epithelial cells. NGS of RNA from inner ear sensory epithelial cells led to the identification of 455 miRNAs in both cochlear and vestibular sensory epithelium, with 30 and 44 miRNAs found in only cochlea or vestibule, respectively. miR-6715-3p and miR-6715-5p were defined for the first time in the inner ear. Gene targets were identified for each of these miRNAs, including Arhgap12, a GTPase activating protein, for miR-6715-3p, implicating this miRNA in sensory hair cell bundle development, actin reorganization, cell adhesion and inner ear morphogenesis.

**Conclusions:**

This study provides a comprehensive atlas of miRNAs in the inner ear sensory epithelia. The results provide further support of the essential regulatory role of miRNAs in inner ear sensory epithelia and in regulating pathways that define development and growth of these cells.

**Electronic supplementary material:**

The online version of this article (doi:10.1186/1471-2164-15-484) contains supplementary material, which is available to authorized users.

## Background

miRNAs play an essential role in inner ear development [1]. miRNAs are small non-coding RNAs that regulate gene expression post-transcriptionally. By binding to sequences in the 3’ untranslated region (UTR) of mRNAs, a miRNA can inhibit target mRNAs by translational suppression and mRNA destabilization [2]. miRNAs have been implicated in hearing loss in humans, since mutations in the seed region of miR-96 or its gene are associated with hearing loss in three extended families [3, 4]. miRNAs were also reported in other human ear pathologies, including an elevation of miR-21 in cholesteatomas [5] and in vestibular schwannomas [6]. miRNAs were found to regulate the otitis media inflammatory response [7]. Their study in humans, however, has been hampered by the unavailability of inner ear RNA from human subjects, making the mouse an invaluable model for studying miRNA development and regulation in the inner ear [8]. In an ENU-induced mutant mouse exhibiting deafness and vestibular dysfunction, a mutation in the miR-96 seed region was found to be responsible for hearing loss, presumably due to the alteration in expression and hence function of both direct and indirect gene targets [9]. A number of mouse mutants affecting miRNA regulation through Dicer have been created, leading to developmental inner ear defects [10, 11]. These conditional knock-out mouse mutants have been instrumental in demonstrating that miRNAs are vital for inner ear morphogenesis and development of the sensory epithelia and sensory neurons.

Expression of over 300 miRNAs have been reported in mouse and rat inner ears thus far, identified by microarray analysis [8, 10, 12, 13], with the majority of work performed on the triad mir-96, −182, and −183 [14]. In order to further identify and characterize miRNAs in the mammalian inner ear, we used NGS for the first time to identify miRNAs in cochlear and vestibular sensory epithelia. Using this method, we identified over 500 inner ear miRNAs, both in common between and unique to each tissue. We further validated and characterized the expression of two miRNAs, miR-6715-3p and miR-6715-5p, in the mouse inner ear. Vezatin, an adherens junctions transmembrane protein previously implicated in deafness [15] and Arhgap12, a Rho GTPase activating protein [16], were verified to be gene targets of miR-6715-3p. Protocadherin 19, a member of the δ2 subclass of nonclustered protocadherins and associated with epilepsy and mental retardation [17], is a gene target of miR-6715-5p. Arhgap12, newly described in the inner ear, is expressed in the epithelial cell-cell junctions of the hair and supporting cells. The miR-6715-3p–Arhgap12 miRNA target pair defines a new regulatory pathway. Understanding these interactions may shed light on known and novel miRNAs, their effect on the development of normal and impaired hearing, and the mechanisms leading towards deafness.

## Results

### RNA-seq derived miRNA transcription profile of the mouse inner ear sensory epithelium

In order to identify miRNAs in the auditory and vestibular sensory epithelium, we sequenced short RNA molecules from cochlear and vestibular sensory epithelia by high–throughput RNA sequencing (RNA-seq). For the cochlea, the sensory epithelium consisted of the organ of Corti, including hair cells, supporting cells, and cells of the greater and lesser epithelial ridges, as well as mesenchymal and neuronal cells. For the vestibule, the sensory epithelium was derived from the saccule, utricle and the lateral and anterior cristae and included hair cells and supporting cells. The advantage of using high–throughput sequencing is that this technique allows for assessment of transcript expression in a given sample at a large dynamic range, from minute quantities to highly abundant ones, and does not require a-priori knowledge for the identification of modified and novel RNA molecules in addition to known ones. cDNA libraries from small RNAs were prepared from dissected sensory epithelium of post-natal day (P)0 mice. Subsequent analysis of sequence reads was done using miRNAkey, a tool for miRNA differential expression analysis [18]. Processed reads were aligned to the mature *Mus musculus* miRNA database (http://www.mirbase.org), allowing one mismatch to occur between a read and the reference, due to isomiRs [19]. Sixty-six and 61.4% of the total reads from cochlear and vestibular sensory epithelia, respectively, were aligned to known mouse miRNAs, indicating a successful isolation of miRNAs. The majority of mapped reads in cochlear and vestibular sensory epithelia, 96.9% in the cochlea and 97.6% in the vestibule, were uniquely mapped to a single miRNA. Overall, 485 and 499 known miRNAs had an RPM (reads per million) mapping higher than 1 in the cochlear and vestibular sensory epithelium, respectively (Additional file 1: Table S1). These lists include all miRNAs that have been studied thus far in the mouse inner ear sensory epithelia, as well as miRNAs that have not been previously detected. Nine miRNAs were most highly expressed in the cochlea and the vestibule, with a similar order in both, except for miR-204-5p, which was the fourth most prevalent in the cochlea and the lowest in the vestibule (Table 1).

**Table 1:** **Most highly expressed miRNAs in cochlear and vestibular sensory epithelium**

miRNA name	preMir	Read count in cochlear sample	Cochlea RPM	Read count in vestibular sample	Vestibule RPM
mmu-miR-182-5p	mmu-mir-182	2421025	453713.969	2746404	485527.023
mmu-miR-181a-5p	mmu-mir-181a	809893	151778.593	661755	116989.320
mmu-miR-26a-5p	mmu-mir-26a	254545	47703.193	243220	42998.001
mmu-miR-204-5p	mmu-mir-204	129332	24237.559	84716	14976.641
mmu-miR-27b-3p	mmu-mir-27b	110796	20763.806	141616	25035.790
mmu-let-7f-5p	mmu-let-7f	86534	16216.968	103956	18378.013
mmu-miR-127-3p	mmu-mir-127	85466	16016.819	131667	23276.942
mmu-miR-22-3p	mmu-mir-22	80333	15054.865	92772	16400.833
mmu-miR-183-5p	mmu-mir-183	79665	14929.678	94421	16692.354

**Figure 1:**
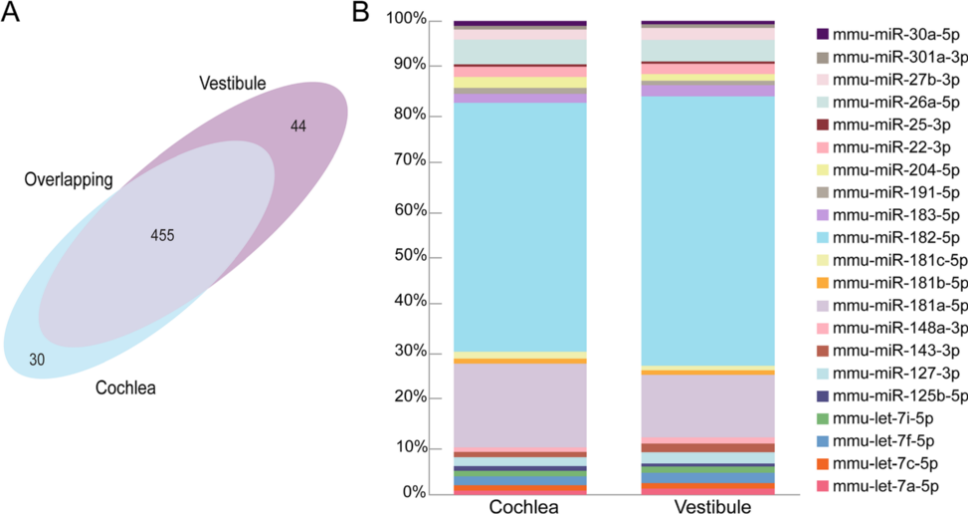
**miRNA expression similarity in the cochlear and vestibular sensory epithelia. A**. Tissue similarity and common expression of inner ear miRNAs. High similarity of miRNA expression in the cochlear and vestibular sensory epithelium can be observed with the majority (455) of the 529 miRNAs, expressed with an RPM > 1, in both tissues. **B**. The relative proportion of each miRNA among the most highly expressed miRNAs in each tissue. The total amount (100%) represents the sum of RPK values of each miRNA. The Y-axis represents the proportion of each miRNA out of this total. Each miRNA is color-coded and indicated in the legend.

Differential expression among the known miRNAs was inferred by miRNAkey, based on the widely-accepted assumption that the number of reads mapped to each miRNA is relative to its level of expression [20, 21]. The reads numbers in each sample were normalized to RPM values, and the relative expression levels of the different miRNAs in the two samples were calculated. In general, miRNA expression was similar in the cochlear and vestibular sensory epithelia. The majority of miRNAs with RPM > 1 were found in both tissues (455, or 86%), with 30 found only in the cochlea and 44 found only in the vestibule (Figure 1A, Additional file 1: Table S1). The rates of the highest expressing miRNAs in each tissue were also highly similar between tissues (Pearson coefficient = 0.99; P < 10^−8^) (Figure 1B). We defined a miRNA as differentially expressed if the fold change between samples was higher than 4 and P < 10^−4^ (Bonferroni corrected). Eleven miRNAs were differentially expressed in cochlear and vestibular sensory epithelia (Table 2).

**Table 2:** **Differentially expressed miRNAs in cochlear and vestibular sensory epithelium**

miRNA name	Read count cochlea	RPM cochlea	Read count vestibule	RPM vestibule	Fold change (1 > 2)	Fold change (2 > 1)	Statistic (Chi^2 dist)	P-value	Corrected P-value (Bonferroni)
mmu-miR-1298-5p	156	29.235	3202	566.070	19.363	0.052	2731.789	0.00E + 00	0.00E + 00
mmu-miR-375-3p	665	124.625	8837	1562.262	12.536	0.080	6909.886	0.00E + 00	0.00E + 00
mmu-miR-211-5p	234	43.853	2696	476.616	10.869	0.092	2026.071	0.00E + 00	0.00E + 00
mmu-miR-1298-3p	16	2.998	116	20.507	6.839	0.146	73.212	0.00E + 00	0.00E + 00
mmu-miR-135b-5p	216	40.480	1059	187.217	4.625	0.216	529.336	0.00E + 00	0.00E + 00
mmu-miR-10b-5p	2584	484.256	12231	2162.275	4.465	0.224	5963.760	0.00E + 00	0.00E + 00
mmu-miR-6240	54	10.120	249	44.020	4.350	0.230	118.741	0.00E + 00	0.00E + 00
mmu-miR-3059-5p	11	2.061	60	10.607	5.145	0.194	32.292	1.33E-08	1.17E-05
mmu-miR-153-3p	933	174.850	175	30.938	0.177	5.652	541.720	0.00E + 00	0.00E + 00
mmu-miR-383-5p	660	123.688	95	16.795	0.136	7.365	437.013	0.00E + 00	0.00E + 00
mmu-miR-6715-5p	78	14.618	12	2.121	0.145	6.890	50.139	1.43E-12	1.27E-09

### Targets for most highly expressed miRNAs

miRNAs and targets exert their function through regulation of biological processes. As each miRNA can have multiple targets, we performed an analysis to discover additional mRNA genes that are computationally predicted to be targets of the three most highly expressed miRNAs, miR-182, miR-181a and miR-26a. Among numerous predictions, a few were of particular interest due to their involvement in auditory function. The targets were defined according to their presence in the list of genes that are associated with deafness in the Mouse Genome Informatics (MGI) (http://www.informatics.jax.org/diseasePortal/) database and predicted by two prediction algorithms, TargetScanMouse and miRanda (Table 3). All miRNA targets presented are involved in auditory function and hearing loss. As a few of the predicted targets overlap, we postulated that there might be underlying regulatory networks prompted by these miRNAs. This was further illustrated using gene ontology (GO) of biological-based processes, performed by geneMANIA (http://www.genemania.org), where 6/16 genes fell in the categories ‘sensory perception of sound’ and ‘sensory perception of mechanical stimulus,’ while 5/16 genes fell in the categories ‘inner ear development’ and ‘ear development’ (Additional file 2: Table S2). These predictions may set the basis for signaling pathways in the inner ear, subject to experimental validation.

**Table 3:** **Predicted mRNA targets for the most abundant miRNAs in the sensory epithelia of the inner ear**

miRNA	Targets
miR-182	Bdnf, Ednrb, Rdx, Rere, Sox2
miR-181a	Chd7, Grid1, Psap, Slc19a2, Tnfrsf11b
miR-26a	Atf2, Fbxo11, Gabrb3, Kcnq4, Rb1, Rere, Slc12a2, Slc19a2

### Identification of inner ear miRNAs

**Figure 2:**
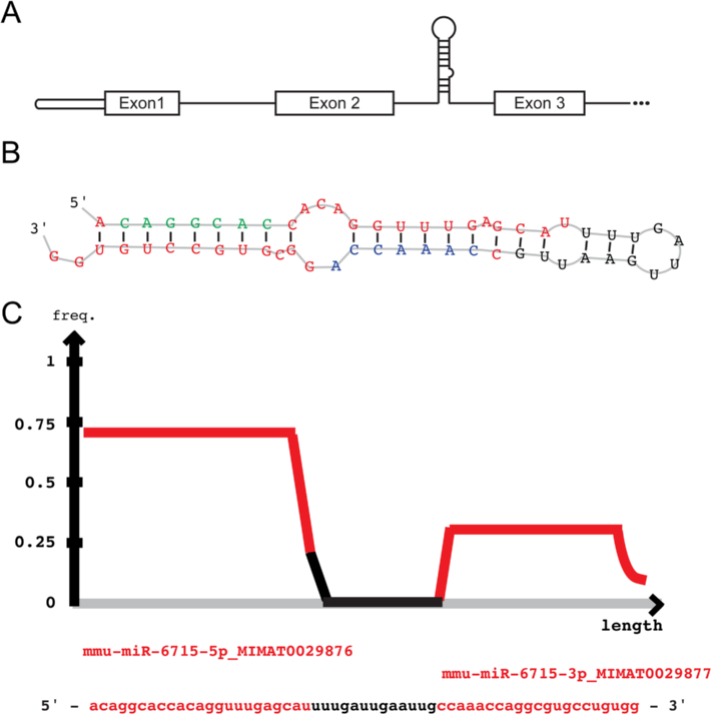
**The genomic location, predicted structure and relative level of expression of miR-6715-3p and miR-6715-5p. A**. pre-miR6715 is located in the second intron of the *Tectb* gene. **B**. miRDeep2 prediction of the RNA secondary hairpin structure of the unprocessed miR-6715-3p, miR-6715-5p, and loop. The seed region of miR-6715-3p is indicated in blue and of miR-6715-5p in green. **C**. Middle density plot shows the distribution of reads in the predicted precursor sequence, as produced by miRDeep2.

In order to identify inner ear miRNAs, the genes associated with “impaired hearing” (MP:0006325) and “deafness” (MP:0001967) were downloaded from MGI (Additional file 3: Table S3). Since these genes are crucial for inner ear development and function, we predicted that miRNAs that reside in their introns may have a role in the inner ear. Out of the hearing associated genes, ten had pri-miRNAs in their introns (Additional file 4: Table S4). One pri-miRNA, expressed in both cochlear and vestibular sensory epithelia with an RPM > 1, was found in the intron of deafness-related gene β-tectorin (*Tectb*) (Figure 2A), which is expressed in the mouse inner ear and associated with altered hearing [22]. This pri-miRNA produces two mature miRNAs, miR-6715-3p and miR-6715-5p (Figure 2B). These miRs were supported by 2.08 and 5.06 reads per million library reads, respectively (Figure 2C). These miRs were also found in an analysis of sequences from mouse data sets derived from different tissues [23]. Provisional ID chr19_45771, available on miRBase (miR-6715; MI0025026), was detected by an extremely low number of reads (0.0021-0.0126 per million library reads) in oocytes, embryonic day (E)7.5 embryo, E15.5 brain, hippocampus, heart, skin, muscle cells, lung and liver by RNA-seq.

### Expression of new inner ear miRNAs

**Figure 3:**
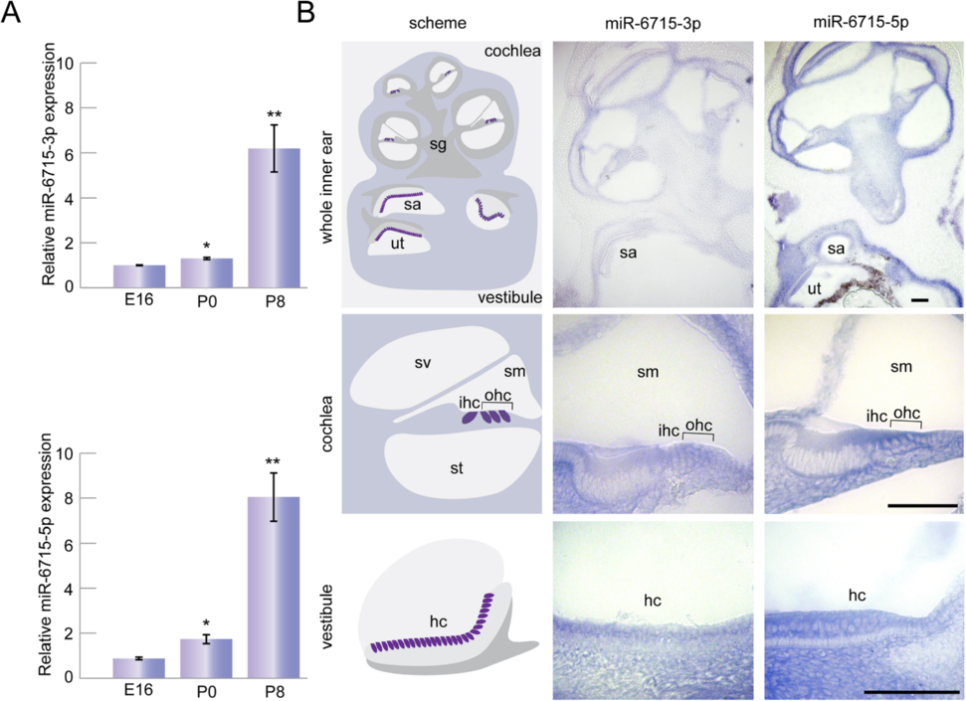
**miR-6715-3p and miR-6715-5p expression in the inner ear. A**. qRT-PCR analysis and validation of novel miRNAs in the mouse cochlea sensory epithelium. miR-6715-3p and miR-6715-5p exhibited increased expression with age between E16 and P8 (*) P < 0.05, (**) P < 0.005. n = 5. **B.** *In situ* hybridization of P0 mouse cochlear and vestibular sections. miR-6715-3p and miR-6715-5p expression was found in the hair and supporting cells of both cochlea and vestibule, spiral and vestibular ganglia, stria vascularis, basilar membrane and in Reissner's membrane. sa, saccule; ut, utricle; sg, spiral ganglia; sm, scala media; st, scala tympani; sv, scala vestibule; ohc, outer hair cells; ihc, inner hair cells; hc, hair cells. Bar: 100 μm.

The expression of miR-6715a-3p and miR-6715a-5p, detected by RNA-seq, was validated by qRT-PCR analysis (Figure 3A). We found both miRNAs to be expressed in the sensory epithelium of the mouse cochlea, at E16, P0 and P8. The expression of both miRNAs was dynamic and increased with age, showing higher expression at P8 compared to P0 and E16, and at P0 compared to E16 (P < 0.005). The two miRNAs were undetected in brain, liver, kidney and heart. This is consistent with the previous RNA-seq detection [23]. The inner ear expression, and the absence or very low expression of the miRNAs in other tested tissues, suggests that they are inner ear-specific miRNAs. The expression prior to the onset of hearing suggests a developmental role for these miRNAs.

In order to explore the spatial expression pattern of the two inner ear miRNAs, *in situ* hybridization analysis was performed on inner ears derived from P0 C57/BL6 mice, at the same age the RNA-seq was performed. Consistent with the qRT-PCR results, expression of the two miRNAs was observed in P0 mice (Figure 3B). A clear staining of both miRNAs was observed in many parts of the inner ear, in spiral and vestibular ganglia, basilar membrane and Reissner's membrane, the stria vascularis and spiral ligament. In a higher magnification, in the cochlea, expression of both miRNAs was observed in the organ of Corti, in the spiral limbus and in particular in the hair and supporting cells. In the vestibular system, staining was observed in sensory epithelia of the saccule and the utricle, in hair and supporting cells.

### Target pathways in auditory and vestibular function

**Figure 4:**
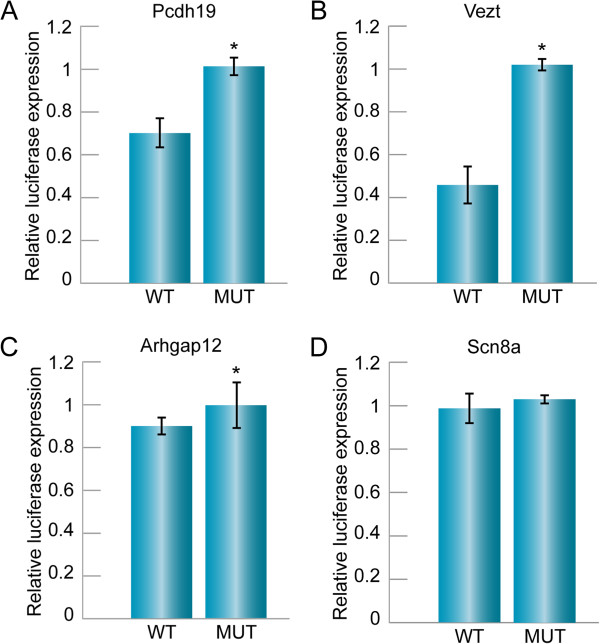
**miRNA-target prediction validation and expression.** Using TargetScan, potential targets of miR- 6715-3p and miR-6715-5p were identified, based on the complimentarily of the seed region and 6-7 nt sequence of the target mRNA. To test the possibility of miR-target regulation, luciferase assays were performed. **A**. Protocadherin19 as a potential target of miR-6715-5p. Wild-type 3’UTR had approximately 65% of luciferase activity as compared to the mutant. (*) P < 0.05. **B**. Vezatin as a potential target of miR-6715-3p. Wild-type 3’UTR had approximately 45% of luciferase activity as compared to the mutant (*) P < 0.05. **C**. Activator for the Rho-type GTPases Arhgap12 as a potential target of miR- 6715-3p. Wild-type 3’UTR had approximately 85% of luciferase activity as compared to the mutant. (*) P < 0.05. **D**. Scn8a is not a direct target for miR-6715-3p, as the luciferase activity was not reduced.

In order to determine what pathway might be impacted by miR-6715-3p and miR-6715-5p regulation, we searched for potential targets. Targets were predicted by TargetScan Custom, which searches for a complementary 3’UTR given a putative seed sequence. miR-6715-3p and miR-6715-5p were each predicted to target 201 and 206 targets, respectively, with ten overlapping targets (Additional file 5: Table S5). We examined which potential targets were inner ear-expressing genes, genes associated with hearing loss in human or mice, or genes encoding proteins involved in pathways relevant for inner ear function. These included protocadherin 19 (Pcdh19) [24] as a candidate target for miR-6715-5p, and vezatin (Vezt) [15], Scn8a [25] and Arhgap12 [16] as candidate targets for miR-6715-3p.To test whether these potential targets have a direct interaction with miR-6715-3p and miR-6715-5p, we examined luciferase activity by inserting the 3’UTR of the potential targets downstream of a luciferase gene. Transfections of these constructs, together with a pre miR-6715 expressing vector into HEK293T cells, and measurement of the relative luciferase activity compared to a mutant 3’UTR construct, were indicative of targeting. Pcdh19 was found to be a direct target of miR-6715a-5p (Figure 4A), with a reduction of luciferase activity by approximately 35%. Vezt and Arhgap12 were found to be direct targets of miR-6715-3p, with a reduction of luciferase activity by 55% and 15%, respectively (Figure 4B, C). Snc8a is not a direct target of miR-6715a-3p, as luciferase activity was not reduced (Figure 4D).

**Figure 5:**
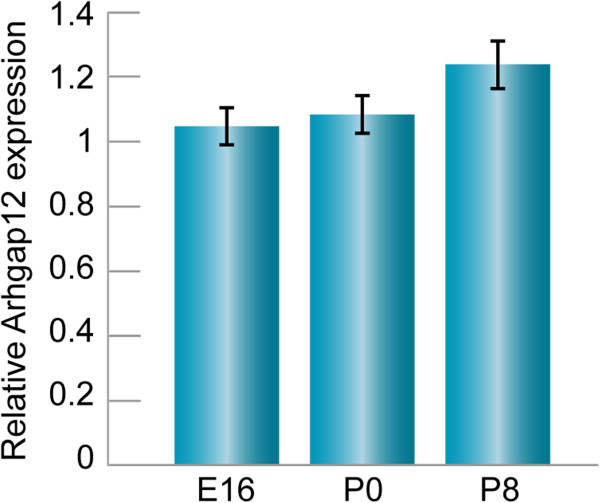
**qRT-PCR analysis of Arhgap12 expression in the mouse cochlea sensory epithelia.** Arhgap12 exhibited a similar expression pattern at E16, P0 and at P8 in cochlear sensory epithelia, with no significant differences between ages. n = 5.

**Figure 6:**
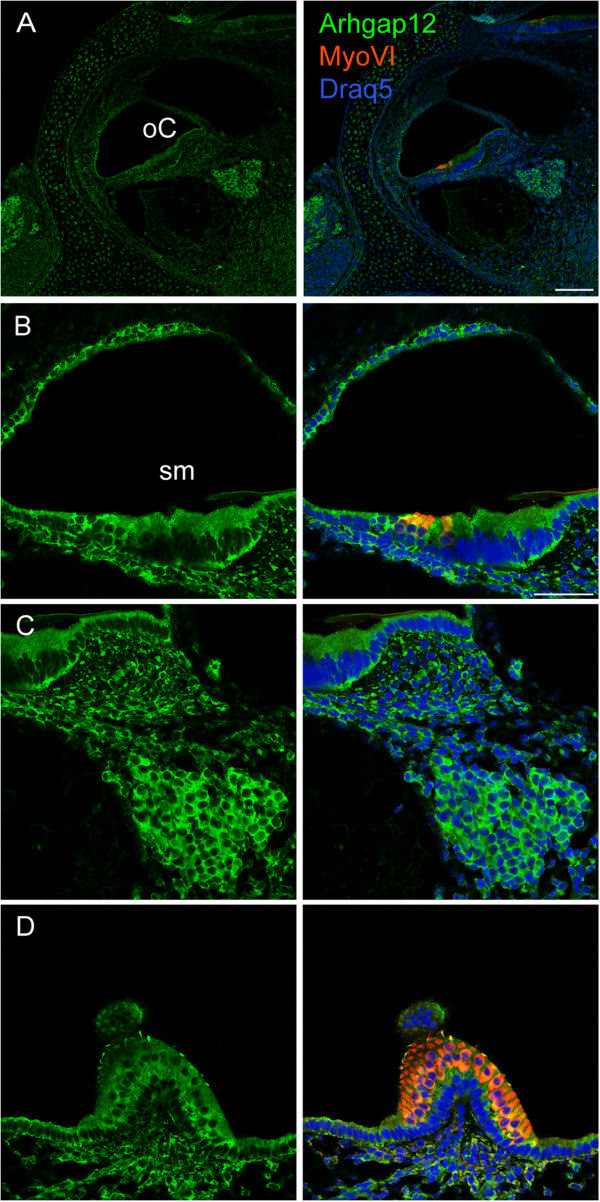
**Arhgap12 protein expression in mouse inner ear sections.** Immunohistochemistry of Arhgap12 (green) in P0 inner ear sections, with hair cells stained by myosin VI (red) and nuclei stained by Draq5 (blue). **A**. Arhgap12 was detected in the organ of Corti, in hair and supporting cells, stria vascularis, spiral ligament and Reissner’s membrane. **B**. Magnification of the organ of Corti, with localization of Arhgap12 in epithelial cells. **C**. Arhgap12 is localized to the spiral ganglia. **D**. In the vestibular system, Arhgap12 is present in hair and supporting cells in the crista. Bar: **A**, 100 μm; **B-D**, 50 μm.

**Figure 7:**
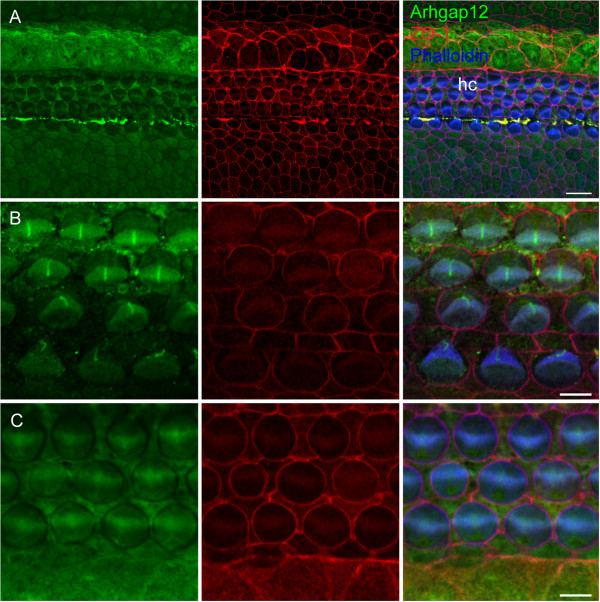
**Arhgap12 protein expression in mouse inner ear whole-mount preparations.** Immunohistochemistry of Arhgap12 (green) in whole mount preparations of P0 mouse cochleas, with cell junctions stained by ZO-1 (red) and actin stained by phalloidin (blue). **A**. Arhgap12 is localized to the hair and supporting cells of the sensory epithelium. **B**. In a higher magnification of A, Arhgap12 is localized to the hair bundle and the kinocilium. **C**. Additional plane showing mutual exclusion of localization of Arhagp12 and ZO-1. hc, hair cells. Bar: **A**, 10 μm; **B**, 5 μm.

Arhgap12 was chosen for further analysis, due to its putative role as a GTPase activating protein (GAPs) for small G proteins and expression in epithelial cell–cell junctions [16]. Temporal expression of *Arhgap12* mRNA was evaluated by qRT–PCR in the cochlear sensory epithelium. In the cochlea, Arhgap12 was expressed at E16, P0 and P8, with a small, though not significant, increase in age (Figure 5). This protein was not previously characterized in the inner ear. We found Arhgap12 expression, using a commercial antibody examined for specificity by peptide competition (Additional file 6) in the organ of Corti, in hair and supporting cells, the stria vascularis, spiral ligament and Reissner’s membrane (Figure 6A, B). The strongest expression was observed in nerve cells, in the spiral ganglia (Figure 6C). In the vestibule, expression was found in the hair and supporting cells (Figure 6D). In whole mount preparations, Arhgap12 was found to be present in hair and supporting cells (Figure 7A-C). Its expression was observed in the same hair cell and cuticular plate planes as ZO-1, but did not overlap (Figure 7A-C). The outer hair cell and Deiter cell junctions expresses ZO-1, while Arhgap12 appears absent in these junctions. Arhgap12 appears in the kinocilium (Figure 6B).

## Discussion

The function of miRNAs and their targets in the mammalian inner ear are now being discovered, as the link between miRNAs and hearing and deafness has been established. The inner ear is a complex tissue with multiple cell types, with miRNAs involved in fine-tuning the multitude pathways required for functional hearing. While there are a relatively small number of miRNAs in mammals, their role in regulation far surpasses their number, since each miRNA can exert its effect on hundreds of downstream targets [26]. Hundreds of miRNAs have been discovered in the inner ear; only a subset of these miRNAs have validated targets (reviewed in Table 1[27]).

Our study was designed to profile the general expression of miRNAs in the inner ear and a comparison of the auditory and vestibular epithelia. We chose the time point of p0 since work by our group [10, 13] and others have demonstrated that miRNAs are expressed and functional at this stage, including miRNA-96, which is essential for hearing [3, 9]. RNA-seq analysis led to the identification of over 500 miRNAs, with miR-182 as the most highly expressed miRNA in both sensory epithelia (Table 1) and accounting for more than 50% of the 20 most highly expressed miRNAs. A few targets have been confirmed for miR-182, including Sox2, Clic5 and Tbx1 [28–30]. The second most abundant miRNA in the cochlear and vestibular sensory epithelia, miR-181a-5p, has no known validated targets in the inner ear. This miRNA has been studied in other systems and found to have a pro-proliferative role in cultured human myeloid leukemia cells [31] and regulates thymic selection in mouse T-cells [32]. The role of this miRNA has been studied in the avian inner ear, where it has been shown to promote proliferation [33].

We validated a number of targets for miR-6715-3p and miR-6715-5p. Predicted targets for the miRNAs described in this study have direct or potential implications for hearing loss. Pcdh19 is expressed in the zebrafish inner ear [24]. Mutations in protocadherin 19 (*PCDH19)* have been associated with X chromosome-linked epilepsy and mental retardation and it is a member of the protocadherin family of proteins [17]. This family includes protocadherin 15 (Pcdh15), with a known function in the tip links of the stereocilia of the inner ear in conjunction with cadherin 23 [34] and has been associated with multiple mutations in both non-syndromic and syndromic hearing loss in humans [35, 36].

Vezatin (Vezt) is an adherens junctions transmembrane protein, and while it is ubiquitously expressed in these junctions, it is especially prevalent in in hair and supporting cells of the mouse inner ear [15]. Vezt mutant mice suffer from late onset progressive hearing loss, due to hair cell apoptosis. Most compelling, the resistance of the organ of Corti to mechanical stress was reduced when vezatin was absent in the inner ears of conditional mutants. These studies led to the prediction that vezation is required to maintain junction integrity and is crucial to protect the junctions from mechanical stress due to sound trauma.

**Figure 8:**
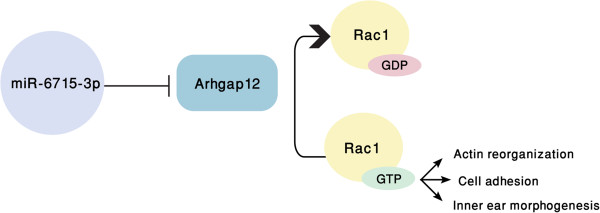
**A newly identified miRNA-target regulatory pathway in the inner ear.** A suggested pathway for miR-6715-3p and Arhgap12, whereby miR-6715-3p down-regulates expression of Arhgap12, which in turn blocks the hydrolysis of Rac1 (Ras-Related C3 Botulinum Toxin Substrate 1) GTPase. This may result in increased cell adhesion, actin reorganization and inner ear morphogenesis.

Arhgap12, a Rho GTPase activating protein (GAP) family member, is a component of cell–cell junctions in kidney epithelial cells [16]. While not previously known to be expressed in the inner ear, its target, Rac1, localizes to hair and supporting cells, specifically in the stereocilia and kinocilium of the hair cells. It was found to be involved in hair cell morphogenesis, specifically in hair bundle development, epithelia actin assembly and cell adhesion. Inactivation of Rac1 specifically in the otic epithelium leads to defects in cochlear morphogenesis, hair bundle orientation and cellular organization of the organ of Corti [37]. Arhgap12 was found to target Rac1 and inactivates it by increasing GTP hydrolysis [38]. Here we suggest a key role for miR-6715-3p in targeting Arhagap12 and in this fashion, influencing Rac1 in the inner ear (Figure 8).

Arhgap12, a GAP protein, is involved in controlling the time period in which Rac1 will remain active. In MDCK cells, Arhgap12 expression overlaps with Rac1 expression [38, 39], probably due to the regulation of Arhgap12 on Rac1 at this time point. In the inner ear, besides the actin-rich areas, Rac1 is localized to hair cells and in particular, the kinocilium [37]. In accordance with our data, Arhgap12, at P0, with expression in the hair cells and kinocilium, might be regulating by Rac1 at this time, when the hair bundle is still developing and needs to be under close regulation. The kinocilium is necessary for proper development [40] and may have a structural role in guiding the architecture of the hair bundle and/or be involved in G-protein signaling in the kinocilium. If the latter is true, then Arhgap12 may regulate its GTPase activity. In our model of the miR-6715-3p circuit in the inner ear, we propose that the miRNA indirectly enhances the action of Rac1 and therefore may promote hair bundle development, actin reorganization, cell adhesion and inner ear morphogenesis.

## Conclusions

The identification of inner ear-related miRNAs by RNA-seq analysis demonstrates that the dataset is reliable not only for characterizing expression profiles of known miRNAs, but also for discovery of novel miRNAs in the inner ear. Further investigation of these miRNAs may shed light on their regulatory roles in various molecular pathways underlying the development of the embryonic inner ear.

## Methods

### RNA isolation

All procedures involving animals were approved and met the guidelines described in the National Institutes of Health Guide for the Use of Laboratory Animals and approved by the Animal Care and Use Committee of Tel Aviv University (M-10-087). Cochlear and vestibular sensory epithelia were dissected from 12 inner ears of 6 P0 C57Bl/6 J mice. The cochlear sensory epithelia included the organ of Corti with some attached membranes, while the vestibular sensory epithelia included the saccule, utricle, lateral crista and anterior crista. The dissected sensory epithelia were placed immediately in Qiazol lysis buffer (Qiagen) on ice and stored at −80°C for several hours. The sensory epithelia was then thawed and total RNA was isolated, using the miRNeasy Mini Kit (Qiagen) without DNAse, which enables purification of total RNA of >18 nt. The isolated RNAs from the samples were precipitated in sodium acetate and glycogen in 80% ethanol (Ambion protocol), in order to further clean and concentrate the RNA samples. The precipitated RNA samples were dissolved in nuclease-free water and their quality was assessed by 1.2% agarose gel (90 V).

### RNA-sequencing

Twelve cochlear epithelia were pooled and twelve vestibular epithelia were pooled, and each pool was used to create a library for MPS and sequenced. No biological repeats were performed. miRNA libraries for MPS were created using the TruSeq SmallRNA SamplePrep Kit (Illumina), with one modification. Instead of running the samples on acrylamide gel in order to isolate the miRNA band, the samples were loaded on a 4% agarose E-Gel and the purification was performed with the Qiagen Kit. The libraries were multiplexed and 2.5 pM of each library was sequenced at SR × 36 bp using the Illumina Genome Analyzer IIx in the same lane at the Functional Genomics Laboratory at Tel Aviv University.

### miRNA sequence analysis

A total of 8,763,589 and 10,048,818 raw sequences of small RNAs were found in the cochlear and vestibular samples, respectively. Small RNA adapter sequences were clipped using fastq-mcf (ea-utils: http://code.google.com/p/ea-utils/), discarding sequences shorter than 16 nucleotides and adapter dimers. Following adapter clipping, 8,340,741 and 9,441,679 reads remained. Differential expression of the resulting sequences was performed using miRNAkey. Specifically, sequence alignment was performed using Burrows-Wheeler Aligner (BWA) [41] against mouse mature miRNA sequences (downloaded from miRBase (Release 20: June 2013; http://www.mirbase.org/), while allowing one mismatch between read and reference. The read count for each read was normalized using the RPM method. Differential expression was tested using chi-squared proportion testing and corrected using the Bonferroni correction for multiple comparisons. Novel miRNA prediction was performed using miRDeep2 [42].

### qRT-PCR

Cochlear sensory epithelia of E16, P0 and P8 C57BL/6 mice was dissected. Small RNAs were extracted using the miRNeasy Mini Kit (QIAGEN). Custom-made probes, designed by Applied Biosystems, were used to detect miRNAs. miRNAs and U6B RNA (endogenous control) were reverse transcribed using the High Capacity cDNA Reverse Transcription Kit (Applied Biosystems). The qRT-PCR reaction was conducted using the FastStart Universal Probe Master (Roche) in the StepOne Plus qRT-PCR machine (Applied Biosystems). All miRNAs expression was normalized to the expression of U6B. At each age, sensory epithelia from 4–5 mice were pooled and five RT-PCR experiments were performed.

### *In situ* hybridization

Inner ears of P0 C57BL/6 mice were dissected and fixed with 4% paraformaldehyde. Whole mount *in situ* hybridization analysis was performed using the Exiqon protocol. miRCURY LNA™ microRNA detection custom-made probes, labeled with digoxygenin (DIG) (Exiqon), were used for the detection of novel miRNAs. Probes were hybridized with the tissue, 20-22°C below Tm of the probe. Probes were detected with the anti-DIG-AP (alkaline phosphatase conjugated) antibody (Roche), and the color reaction was developed using the NTB/BCIP (Sigma). The ears were frozen and cryosectioned to 10-18 μm sections and mounted. Images were taken using the Zeiss Aviovert200 M microscope. Three experiments were performed, and 3–5 ears were included in each experiment.

### miRNA target prediction

Target prediction for all miRNAs was predicted by TargetScan Custom (Release 5.2; http://www.targetscan.org/vert_50/seedmatch.html), TargetScanMouse (Release 5.2, http://www.targetscan.org/mmu_50/) and/or miRanda (http://www.microrna.org/microrna/home.do). Chosen potential targets were compared to gene expression databases (SHIELD; https://shield.hms.harvard.edu/) and known hearing loss genes and loci (Hereditary Hearing Loss Homepage, http://hereditaryhearingloss.org).

### Luciferase assay

3’ UTRs of chosen potential targets were cloned into the pGL3 luciferase reporter vector, downstream to the luciferase reporter gene, creating the 3’UTR-wt-pGL3 vector. The first three nucleotides of the miRNA binding site in the 3’UTR were mutated by site directed mutagenesis, creating the 3’UTR-mut-pGL3 vector. The miR-6715 miRNA expression vector was created by cloning the genomic polycistronic miR-6715-3p and miR-6715-5p into the miRvec expression vector (obtained as a gift from Reuven Agami) [43]. HEK293T cells were transiently transfected with either 3’UTR-wt-pGL3 or with 3’UTR-mut-pGL3, for each of the tested targets, together with the corresponding miRNA expression vector and Renilla expressing vector. The luciferase reporter assay was performed 48 hours following transfection using the Dual-Luciferase® Reporter assay system (Promega). Three experiments were conducted, with duplicates.

### Immunohistochemistry

Whole mount and paraffin sections of P0 C67/BL6 mouse inner ear were prepared for staining as previously described [44]. Myosin VI was used to stain hair cells, phalloidin to stain actin and Draq5 to stain nuclei. The following antibodies were used: goat-anti-Arhgap12 antibody (Santa Cruz), 1:100; mouse-anti-ZO-1 (Zymed), 1:100; rabbit-anti-myosin VI (Proteus BioSciences) 1:200; and Draq5 1:600 (Abcam). Antibody specificity of Arhgap12 was assessed by a competition assay in HCT116 cells, with blocking by an Arhagap12 peptide (Santa Cruz) (Additional file 6).

### Statistics

For qRT-PCR and luciferase assays, the Student’s two-tailed *t* test P values of less than 0.05 were considered to be statistically significant and those of less than 0.005 were considered to be highly statistically significant. The data in the figures are presented by mean + SEM. Differential expression of miRNAs between paired samples was measured using a chi-squared statistic. *P*-values are calculated for the null hypothesis of no differential expression between the two samples. Final *P*-values were corrected using the Bonferroni correction for multiple hypotheses testing [18].

## Availability of supporting data

The RNA-seq data from this study is available in the NCBI Sequence Read Archive (SRA) (http://www.ncbi.nlm.nih.gov/sra), under accession number SRP043019.

## Electronic supplementary material

Additional file 1: Table S1: miRNAkey output table for cochlear vs. vestibular miRNA differential expression. miRNA differential expression analysis includes the read count, RPM, fold change and comparison statistics for each miRNA transcript. (XLS 137 KB)

Additional file 2: Table S2: Predicted targets categorized by gene ontology (GO) of biological-based processes. (DOCX 63 KB)

Additional file 3: Table S3: Genes associated with “impaired hearing” and “deafness” impairment in MGI. (XLS 33 KB)

Additional file 4: Table S4: Expression of pri-miRNAs found inside introns of “impaired hearing” and “deafness” genes, as defined in MGI. (XLS 27 KB)

Additional file 5: Table S5: Predicted targets for miR-6715-3p and miR-6715-5p. Source: TargetScanHuman Custom Release 5.2: June 2011. (XLSX 15 KB)

Additional file 6: **Specificity of the Arhgap12 antibody was tested by a peptide competition assay.** HCT116 cells were stained with an Arhgap12 antibody and an Arhgap12 peptide. No staining of Arhgap12 was observed in the blocked cells. Bar: 25 μm. (TIFF 2 MB)
