# Supplementary material for: Next-generation sequencing of small RNAs from inner ear sensory epithelium identifies microRNAs and defines regulatory pathways
Source: BMC Genomics. 2014 Jun 18;15(1):484. doi: 10.1186/1471-2164-15-484 (PMC4073505; doi:10.1186/1471-2164-15-484)
Supplement: Supplementary file 2 — Additional file 2: Table S2: Predicted targets categorized by gene ontology (GO) of biological-based processes. (DOCX 63 KB) [file 12864_2014_6165_MOESM2_ESM.docx]

**Table S2. Predicted targets categorized by gene ontology (GO) of biological-based processes**

| Gene symbol | Gene name | ‘Inner ear development' and 'ear development' | ‘Sensory perception of sound' and 'sensory perception of mechanical stimulus' |
| --- | --- | --- | --- |
| *CHD7* | chromodomain helicase DNA binding protein 7 | **X** | **X** |
| *GRID1* | glutamate receptor, ionotropic, delta 1 |  |  |
| *PSAP* | prosaposin |  |  |
| *SLC19A2* | solute carrier family 19 |  |  |
| *TNFRSF11B* | tumor necrosis factor receptor superfamily, member 11b |  |  |
| *BDNF* | brain derived neurotrophic factor | **X** |  |
| *EDNRB* | endothelin receptor type B |  |  |
| *RDX* | radixin |  |  |
| *RERE* | arginine glutamic acid dipeptide (RE) repeats |  |  |
| *SOX2* | SRY-box containing gene 2 | **X** | **X** |
| *ATF2* | activating transcription factor 2 |  |  |
| *FBXO11* | F-box protein 11 |  | **X** |
| *GABRB3* | gamma-aminobutyric acid (GABA) A receptor, subunit beta 3 | **X** | **X** |
| *KCNQ4* | potassium voltage-gated channel, subfamily Q, member 4 | **X** | **X** |
| *RB1* | retinoblastoma 1 |  |  |
| *SLC12A2* | solute carrier family 12, member 2 |  | **X** |
