# Supplementary figures and images for: Next-generation sequencing of small RNAs from inner ear sensory epithelium identifies microRNAs and defines regulatory pathways
Source: BMC Genomics. 2014 Jun 18;15(1):484. doi: 10.1186/1471-2164-15-484 (PMC4073505; doi:10.1186/1471-2164-15-484)

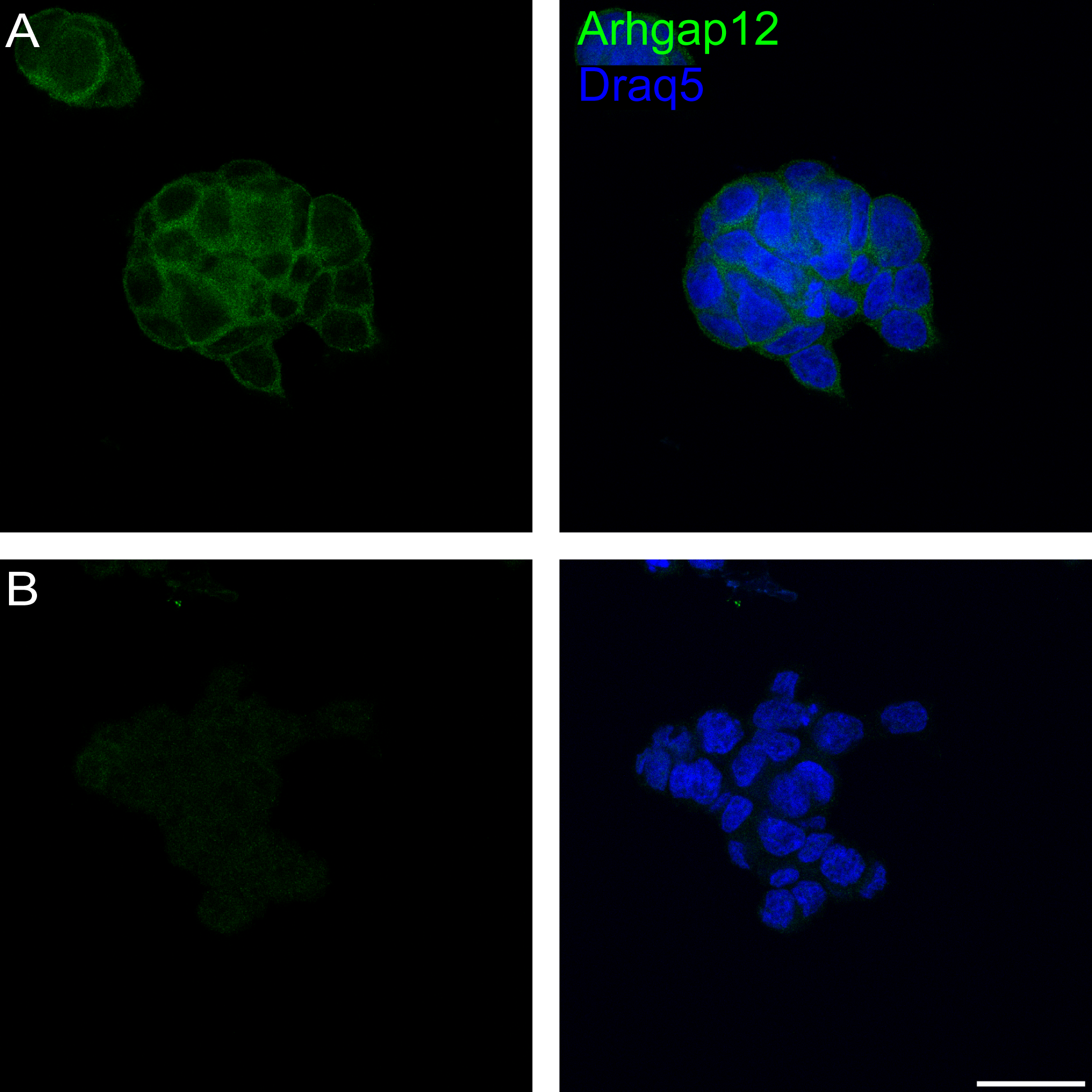

Supplement: Supplementary file 6 — Additional file 6: Specificity of the Arhgap12 antibody was tested by a peptide competition assay. HCT116 cells were stained with an Arhgap12 antibody and an Arhgap12 peptide. No staining of Arhgap12 was observed in the blocked cells. Bar: 25 μm. (TIFF 2 MB) [file 12864_2014_6165_MOESM6_ESM.tiff]
